# Sustained and Long-Term Release of Doxorubicin from PLGA Nanoparticles for Eliciting Anti-Tumor Immune Responses

**DOI:** 10.3390/pharmaceutics14030474

**Published:** 2022-02-22

**Authors:** Jeongrae Kim, Yongwhan Choi, Suah Yang, Jaewan Lee, Jiwoong Choi, Yujeong Moon, Jinseong Kim, Nayeon Shim, Hanhee Cho, Man Kyu Shim, Sangmin Jeon, Dong-Kwon Lim, Hong Yeol Yoon, Kwangmeyung Kim

**Affiliations:** 1 KU-KIST Graduate School of Converging Science and Technology, Korea University, 145 Anam-ro, Seongbuk-gu, Seoul 02841, Korea; 218312@kist.re.kr (J.K.); cyhwill@noxpharm.co.kr (Y.C.); haehwan@kist.re.kr (S.Y.); 220343@kist.re.kr (J.L.); 217802@kist.re.kr (J.C.); 218843@kist.re.kr (J.K.); sny3766@kist.re.kr (N.S.); dklim@korea.ac.kr (D.-K.L.); 2 Center for Theragnosis, Biomedical Research Institute, Korea Institute of Science and Technology (KIST), Seoul 02792, Korea; phoenix0310@kist.re.kr (Y.M.); ricky@kist.re.kr (H.C.); mks@kist.re.kr (M.K.S.); jeon@kist.re.kr (S.J.); seerou@kist.re.kr (H.Y.Y.)

**Keywords:** cancer, chemoimmunotherapy, immunogenic cell death, nanomedicine, immune checkpoint blockade

## Abstract

Immunogenic cell death (ICD) is a powerful trigger eliciting strong immune responses against tumors. However, traditional chemoimmunotherapy (CIT) does not last long enough to induce sufficient ICD, and also does not guarantee the safety of chemotherapeutics. To overcome the disadvantages of the conventional approach, we used doxorubicin (DOX) as an ICD inducer, and poly(lactic-co-glycolic acid) (PLGA)-based nanomedicine platform for controlled release of DOX. The diameter of 138.7 nm of DOX-loaded PLGA nanoparticles (DP-NPs) were stable for 14 days in phosphate-buffered saline (PBS, pH 7.4) at 37 °C. Furthermore, DOX was continuously released for 14 days, successfully inducing ICD and reducing cell viability in vitro. Directly injected DP-NPs enabled the remaining of DOX in the tumor site for 14 days. In addition, repeated local treatment of DP-NPs actually lasted long enough to maintain the enhanced antitumor immunity, leading to increased tumor growth inhibition with minimal toxicities. Notably, DP-NPs treated tumor tissues showed significantly increased maturated dendritic cells (DCs) and cytotoxic T lymphocytes (CTLs) population, showing enhanced antitumor immune responses. Finally, the therapeutic efficacy of DP-NPs was maximized in combination with an anti-programmed death-ligand 1 (PD-L1) antibody (Ab). Therefore, we expect therapeutic efficacies of cancer CIT can be maximized by the combination of DP-NPs with immune checkpoint blockade (ICB) by achieving proper therapeutic window and continuously inducing ICD, with minimal toxicities.

## 1. Introduction

Immunogenic cell death (ICD) is representative of induced antitumor immunity, and in practice various cancer therapies could elicit immunogenicity by initiating ICD [1,2,3]. For instance, it has become known that several chemotherapeutics such as doxorubicin (DOX) kill cancer cells directly as well as cause successive antitumor immune responses by exposure to damage-associated molecular patterns (DAMPs) such as calreticulin (CRT), high mobility group box 1 (HMGB1), and adenosine triphosphate (ATP) [4,5,6]. Due to the ICD inducers, dendritic cells (DCs) subsequently act as a bridge between innate and adaptive immunity by presenting tumor antigens to T cells [7,8], leading to strong antitumor immunity based on the more immune-friendly reverted environment [9,10]. In spite of the key role of chemotherapeutics as potent inducers of ICD, their use has been severely limited for several reasons, such as poor pharmacokinetics (PK), high toxicity, and off-target effects [11]. Nanotechnology-based drug delivery systems (Nano-DDS) have been developed to overcome these traditional issues of cytotoxic drugs, and have enabled the prolonged circulation and increased tumor accumulation of drugs, and simultaneously reduced treatment-related toxicity [12,13,14]. Despite these promising signs, there still remain a few hurdles to be surmounted in nanomedicine, for example low delivery efficiency (<1%), heterogeneity of drug accumulation, and inadequate toxicokinetics causing side effects and immune adverse effects [15,16,17,18].

Nano-DDS-based local delivery could be a good alternative to solve most of these existing problems. Based on the nano-DDS, cytotoxic drugs could be relatively uniform and effective localized delivery at the target tumor sites, with minimal toxicities and controllable drug kinetics even elsewhere in the body [19,20,21]. Specifically, the effective and safe cancer therapy can be sufficiently provided by the optimal dose range of the nanomedicine inducing continuous ICD [22,23], especially based on the preserved tumor microenvironment (TME) with minimal histotoxicity and immunotoxicity [24,25,26,27]. Especially, immune checkpoint blockade (ICB) functions as a tumor suppressor by disrupting the existing interactions between immune cells and tumor cells [28]. ICB restores suppressed T cell-mediated antitumor immunity [29,30], and reverts tumor immune microenvironment (TIME) [31]. Therapeutic efficacies against cancer can be maximized in the responsive TIME [32]. There is no doubt of the promising potentials and therapeutic outcomes unleashing the strong antitumor immune responses in chemotherapy-based cancer immunotherapy [33,34,35,36,37].

In this regard, we hypothesized that ICD might be sufficiently induced during the whole treatment using poly(lactic-co-glycolic acid) (PLGA)-based nanomedicine by controlling the release kinetics of DOX. PLGA is a well-known biodegradable polymer, and has been widely used as a nanocarrier for controlled release of drug in the treatment of cancer [38,39]. The degradation rate and drug release kinetics of PLGA-based nanomedicine can be affected by its intrinsic polymer characteristics, such as the lactide:glycolide (L:G) ratio and the molecular weight [40,41,42]. Thus, PLGA platform is very suitable for controlling the efficacy and toxicity of drugs with the adequate drug loading and the desired controlled drug release profiles.

Tumor growth might be strongly inhibited, even in repeated treatment of DOX-loaded PLGA nanoparticles (DP-NPs) with reduced toxicities. The undevastated environment might fully promote antitumor immune responses, leading to high therapeutic performance, especially with the combination of ICB. To demonstrate our hypothesis, DOX was encapsulated with PLGA NPs using an oil-in-water (o/w) method, as we previously reported [43]. The morphology was examined using field emission scanning electron microscopy (FE-SEM). The efficiency of drug loading inside of NPs was measured using UV–vis spectroscopy. Size distribution, surface charge, and stability were determined using a zeta-sizer. Release kinetics of DOX were monitored under physiological conditions. Cellular uptake was observed using a confocal microscopy. Cytotoxicity was evaluated using cell counting kit-8 (CCK-8). ICD-associated DAMPs were investigated in cancer cell cultured system. All these features and behaviors of DP-NPs were characterized in vitro, and the other studies were performed in vivo. In vivo release kinetics of DOX from DP-NPs were monitored in CT26 tumor-bearing mice. Tumor growth inhibition by DP-NPs, also in combination therapy with anti-PD-L1 (aPD-L1) blockade, was monitored for 4 weeks. Finally, histology and immune cell analyses were performed simultaneously.

## 2. Materials and Methods

### 2.1. Materials

PLGA with acid endcaps (LG 50:50, Mn 5–10 kDa) was obtained from PolySciTech (West Lafayette, IN, USA). DOX hydrochloride (DOX-HCl) was obtained from FutureChem (Seoul, Korea). *N*,*N*-diisopropylethylamine (DIPEA), chloroform, dichloromethane (DCM), poly(vinyl alcohol) (PVA, hydrolyzed, Mw 30–70 kDa), dimethyl sulfoxide (DMSO), fluorescein amine, *N*-ethyl-*N*′-(3-(dimethylamino)propyl)carbodiimide (EDC), and *N*-hydroxysuccinimide (NHS) were obtained from Sigma-Aldrich (St. Luis, MO, USA). Dialysis membranes (MWCO 3.5 kDa and 12–14 kDa) were purchased from Repligen Corporation (Waltham, MA, USA). CCK-8 was purchased from Dojindo (Rockville, MD, USA). ATP assay kit was purchased from Beyotime (Shanghai, China). Anti-CRT (mouse, AF647) mAb, anti-HMGB1 (mouse) pAb, and hematoxylin and eosin (H&E) staining were purchased from Abcam (Cambridge, UK). Terminal deoxynucleotidyl transferase (TdT) dUTP nick end labeling (TUNEL) assay kit was purchased from Promega (Madison, WI, USA). Amicon^®^ centrifugal filter unit (MWCO 10 kDa) was purchased from Merk Millipore (Burlington, MA, USA). Tumor dissociation kit (mouse) was obtained from Miltenyi Biotec (Bergisch Gladbach, Germany). Anti-CD16/CD32 (mouse, Fc Block^TM^) was purchased from BD Biosciences (San Jose, CA, USA). Anti-CD11c and anti-CD8a (mouse, APC), anti-CD40 (mouse, PE), anti-CD86 and anti-CD3 (mouse, FITC), and anti-CD45.2 (mouse, PE-Cy7) Abs were obtained from BioLegend (San Diego, CA, USA). aPD-L1 Ab (mouse) was purchased from BioXCell (Lebanon, NH, USA). CT26 (mouse colon carcinoma) was obtained from the Korean Cell Line Bank (Seoul, Korea). RPMI-1640 and fetal bovine serum (FBS) were obtained from Welgene Inc. (Gyeongsangbuk-do, Korea).

### 2.2. Preparation of DP-NPs

To prepare hydrophobic DOX, 100 mg of DOX-HCl was dissolved in 10 mL of deionized (DI) water and 200 µL of DIPEA was added additionally for 1 h. The desalted DOX was obtained after extraction with chloroform and subsequent evaporation. DP-NPs were prepared by the o/w single-emulsion solvent evaporation method. Briefly, 20 mg of PLGA was dissolved in 0.4 mL of DCM. Ten milligrams of desalted DOX dissolved in 0.4 mL of DCM was added into PLGA solution. For reference, to prepare PLGA NPs (P-NPs) as a vehicle, 0.4 mL of DCM without DOX was added into PLGA solution. Next, 3 mL of aqueous PVA solution (3 wt%) was slowly added into the previous mixture. This mixture was emulsified by sonication (180 watts) for 45 s in an ice bath, and stirred (800 rpm) to remove DCM for 3 h at room temperature (RT) after the addition of 6 mL of distilled water (DW). Then the suspension was centrifuged for 15 min at 15,000 rpm and washed twice with 30 mL of DW, subsequently lyophilized for 2 days after 0.45 μm filtration to obtain DP-NPs. To monitor cellular uptake of DP-NPs in vitro, fluorescein amine was chemically conjugated to carboxylic acid on the end of PLGA in the presence of EDC and NHS. In brief, 100 mg of PLGA was dissolved in 10 mL of DMSO, and mixed with 1 mL of DMSO containing 5.5 mg of EDC and 3.3 mg of NHS. Then, 1 mL of DMSO containing 10 mg of fluorescein amine was slowly added into the PLGA solution and stirred for 12 h at RT. The resulting solution was purified using a dialysis membrane (MWCO 3.5 kDa) against DMSO:DW (1:0, 1:1, 0:1 v/v%) for 3 days. The fluorescein-labeled PLGA (FITC-PLGA) was obtained after the lyophilization for 2 days. Finally, FITC-labeled DP-NPs were formulated as described above using FITC-PLGA and PLGA at 1:9 wt%. The DOX contents in DP-NPs and FITC-labeled DP-NPs were confirmed using a UV–vis spectrophotometer before use.

### 2.3. In Vitro Formulation and Characterization of DP-NPs

The morphology of DP-NPs was observed using FE-SEM (Regulus 8230, Hitachi, Tokyo, Japan). P-NPs and DP-NPs were dispersed in the DI water (0.2 mg/mL) for measurement of size distribution and surface charge. These features were measured using a zeta-sizer (Nano-ZS, Malvern Instruments, London, UK). The drug loading efficiency of DOX in DP-NPs was measured using UV–vis spectroscopy (Cary 60 UV–vis spectrophotometer, Agilent Technologies, Santa Clara, CA, USA), absorbance at 480 nm. The absorbance of DP-NPs (1 mg of DOX in 1 mL of DW) was measured at 480 nm, and calculated based on the calibration curve of DOX in various concentration and the baseline correction. The serum stability of P-NPs and DP-NPs were monitored in condition of 10% (v/v) of FBS-containing phosphate-buffered saline (PBS, pH 7.4) at 37 °C for 2 weeks. The size of both NPs was measured every 2 days using a zeta-sizer. Similarly, the release profile of DOX from DP-NPs, in 0.1% Tween 80-containing PBS (pH 7.4), was monitored in the water bath shaker (100 rpm) at 37 °C for 2 weeks. The DP-NPs (10 mg of DOX) initially within the dialysis membrane (MWCO 12–14 kDa) were dispersed in 10 mL of PBS, continually replaced as fresh PBS every other day. The amount of contained DOX in the harvested PBS was analyzed using UV–vis spectroscopy, absorbance at 480 nm.

### 2.4. In Vitro Cellular Uptake and Cytotoxicity

Cellular uptake imaging of FITC-labeled DP-NPs was performed in the CT26 tumor cells using a confocal microscopy (Leica TCS SP8, Wetzlar, Germany). Briefly, the CT26 tumor cells (1 × 10^5^ cells) were seeded into dishes and incubated at 37 °C for 24 h. Then, the medium in dishes was replaced as 2 mL of new medium containing 2 µM of DOX-HCl or 10 µg of FITC-labeled DP-NPs (equiv. of 2 µM of DOX-HCl). The CT26 tumor cells were incubated for more 48 h, and they were washed with PBS (pH 7.4). Then, they were fixed with a paraformaldehyde (PFA) solution (4% in PBS) for 15 min. 4′,6-diamidino-2-phenylindole (DAPI) was utilized for nuclear staining at RT for 15 min. Cells were observed using a confocal microscopy equipped with diode, Ar, and HeNe lasers at 405, 488/514, and 633, respectively. Cell viability of the CT26 tumor was measured using CCK-8. The CT26 tumor cells (5 × 10^3^ cells) were seeded into 96-well plates and incubated at 37 °C for 24 h. Cells were treated with various concentration (from 0.001 to 10 μM) of DOX-HCl and the equivalent DP-NPs, and further incubated for more 48 h. They were washed with PBS (pH 7.4) and incubated with a CCK-8 reagent-containing medium for 30 min. The absorbance at 450 nm of each medium was measured using a tunable microplate reader (VersaMax^TM^, Molecular Devices, San Jose, CA, USA). For reference, the cell viability of the vehicle-treated CT26 tumor was also measured, in the same way as DP-NPs.

### 2.5. In Vitro Analysis of ICD-Associated DAMPs

To analyze the induced ICD in CT26 tumor cells, we observed several molecular markers of DAMPs such as CRT, HMGB1, and ATP. For reference, the analysis with the vehicle-treated group was also performed in each experiment, in the same way as DP-NPs. CRT exposed on the surface of CT26 tumor cells was observed using a confocal microscopy. Then, the medium in dishes was replaced as 2 mL of new medium containing 2 µM of DOX-HCl or 10 µg of DP-NPs (equiv. of 2 µM of DOX-HCl). The CT26 tumor cells were incubated for more 48 h, and they were washed with PBS (pH 7.4) and fixed with a PFA solution (4% in PBS) for 15 min. Cells were treated with the bovine serum albumin (BSA) blocking buffer (5% in PBS) to prevent the non-specific binding for 10 min. Then they were stained with CRT Ab for 1 h, and washed with PBS (pH 7.4). DAPI was utilized for nuclear staining at RT for 15 min. The stained CT26 tumor cells were observed using a confocal microscopy. Intensities of CRT was quantified using Image-Pro Plus software (Media Cybernetics, Silver Spring, MD, USA). The released HMGB1 and the secreted ATP in CT26 tumor cell culture medium was analyzed by Western blotting and ATP assay, respectively. Briefly, the CT26 tumor cells (1 × 10^6^ cells) were seeded into culture dishes and incubated at 37 °C for 24 h. Then, the medium in dishes was replaced as the new medium (2 µM of DOX-HCl and the equivalent DP-NPs). The CT26 tumor cells were incubated for more 48 h. Their culture medium was collected and separated into two parts by centrifugation using a filter unit. The filtered sample (<10 kDa) was used to measure ATP levels, and the other sample (>10 kDa) was used to observe HMGB1 expressions. Band intensities of HMGB1 were quantified using ImageJ software (NIH, Bethesda, MD, USA).

### 2.6. In Vivo Drug Release Kinetics

All experiments with specific pathogen-free (SPF) mice were performed in compliance with the relevant laws and institutional guidelines of Korea Institute of Science and Technology (KIST), and Institutional Animal Care and Use Committee (IACUC) approved the experiments (approval number: KIST-2020-070, 27/05/2020). Six-week-old male BALB/c mice were purchased from OrientBio Inc. (Gyeonggi-do, Korea). The release of DOX from DP-NPs were observed by its fluorescence in CT26 tumor-bearing mice after the intratumoral injection of DP-NPs for 2 weeks. For reference, a DOX-HCl-treated mouse was also monitored as a control. Briefly, mice were subcutaneously injected with CT26 tumor cells (1 × 10^6^ cells/60 μL) on their left flank. When the tumor volume reached 50–80 mm^3^, 0.25 mg of DOX-HCl (10 mg/kg of DOX, DOX 10), 0.5 mg of DP-NPs (equiv. of 5 mg/kg of DOX, DP-NPs 5), 1 mg of DP-NPs (equiv. of 10 mg/kg of DOX, DP-NPs 10), and 1 mg of DP-NPs with two injections at intervals of 10 days (DP-NPs 10 × 2, equiv. of 10 mg/kg of DOX per injection) were intratumorally injected after dispersed using 30 μL of sterilized PBS (pH 7.4). Near-infrared fluorescence (NIRF) images were acquired by In Vivo Imaging System (IVIS, Lumina Series III, PerkinElmer, Waltham, MA, USA), and the fluorescence from these images were quantified using Living Image software (PerkinElmer, Waltham, MA, USA).

### 2.7. In Vivo Tumor Growth Inhibition and Toxicity

Mice were subcutaneously injected with CT26 tumor cells (1 × 10^6^ cells/60 μL) on their left flank. When the tumor volume reached 50–80 mm^3^, CT26 tumor-bearing mice were treated with PBS, P-NPs, DOX-HCl (10 mg/kg), DP-NPs (5, 10, 10 × 2 mg/kg), aPD-L1 Ab (10 × 5 mg/kg)-combined DOX-HCl (10 mg/kg) and DP-NPs (10 × 2 mg/kg). All samples were prepared using 30 μL of sterilized PBS (pH 7.4). Tumor growth, body weight change, and survival of each group were monitored for 4 weeks. Tumor volumes (calculated as 0.52 × Diameter_Longitudinal_ × Diameter_Transverse_^2^) and body weight were measured every two days. We limited the maximum allowable size of subcutaneous tumors in control groups, and mice with a tumor size 3000 mm^3^ were counted statistically dead, for survival. Photographs of spleens were obtained on the 14th day, and the weight of the spleen was also measured to examine the DOX-induced systemic toxicity.

### 2.8. In Vivo ICD-Associated DAMPs and Immune Cell Analysis

On the 14th day after the first injection, CT26 tumor tissues which were directly treated with PBS, P-NPs, DOX 10, and DP-NPs (5, 10, 10 × 2) and tumor-draining lymph nodes (TDLNs) were excised from the CT26 tumor-bearing mice to analyze ICD-associated DAMPs and immune cell population in the tumor tissues. The tumor tissues were dissociated with the tumor dissociation kit at 2% (v/v) of FBS-containing PBS, and centrifuged at 500 g for 10 min. The supernatant was harvested for Western blotting analysis of HMGB1, and the cells were collected for flow cytometric analysis of CTLs. The cells in TDLNs were also collected and centrifuged similarly for flow cytometric analysis of DCs. Red blood cell (RBC) lysis and single-cell suspension were performed for staining with the adequate Abs. FcR blocking reagent was used to eliminate non-specific staining. Then, the single cells from TDLNs were fluorescently stained with CD45, CD11c, CD40, and CD86 Abs for identifying maturated DCs, and those from tumor tissues were stained with CD45, CD3, and CD8 Abs for identifying CTLs. After the incubation for 1 h, harvested cells were characterized by a flow cytometer (CytoFLEX, Beckman Coulter, CA, USA). These results were analyzed using FlowJo^TM^ software (BD Biosciences, NJ, USA).

### 2.9. TUNEL Assay and H&E Staining

Each sample was collected on the 14th day after the first treatment of PBS, P-NPs, DOX-HCl (10 mg/kg), and DP-NPs (5, 10, 10 × 2 mg/kg) in the previously stated in vivo experiments for TUNEL assay and H&E staining. TUNEL assay was performed to assess DOX-induced apoptotic changes in tumor tissues. The apoptotic cells were determined by TUNEL assay kit (Promega, Madison, WI, USA). Briefly, tumor tissues were deparaffinized and fixed with a PFA solution (4% in PBS) for 15 min. Proteinase K solution, equilibration and incubation buffers were sequentially added after the tissue permeabilization. Reaction was stopped in an hour, and the subsequent stains were performed with propidium iodide and DAPI. The localized green fluorescence of apoptotic cells in prepared samples were observed using a confocal microscopy. H&E staining was also performed in the tissues of tumor and major organs (spleen, liver, lung, kidney, and heart) to further investigate DOX-induced in vivo toxicities. Briefly, the deparaffinized and rehydrated tumor tissues were sequentially stained with hematoxylin and eosin. After the dehydration, stained tumor tissues sliced into 10 μm sections were observed using an optical microscopy.

### 2.10. Statistical Analysis

In this study, one-way analysis of variance (ANOVA) was performed for a comparison of several experimental groups using Origin 2020 software (OriginLab Corporation, Northampton, MA, USA). Statistical significance was marked with an asterisk (*p < 0.05, **p < 0.01, and ***p < 0.001) in the figures.

## 3. Results and Discussion

### 3.1. Characterization of the DP-NPs

**Figure 1:**
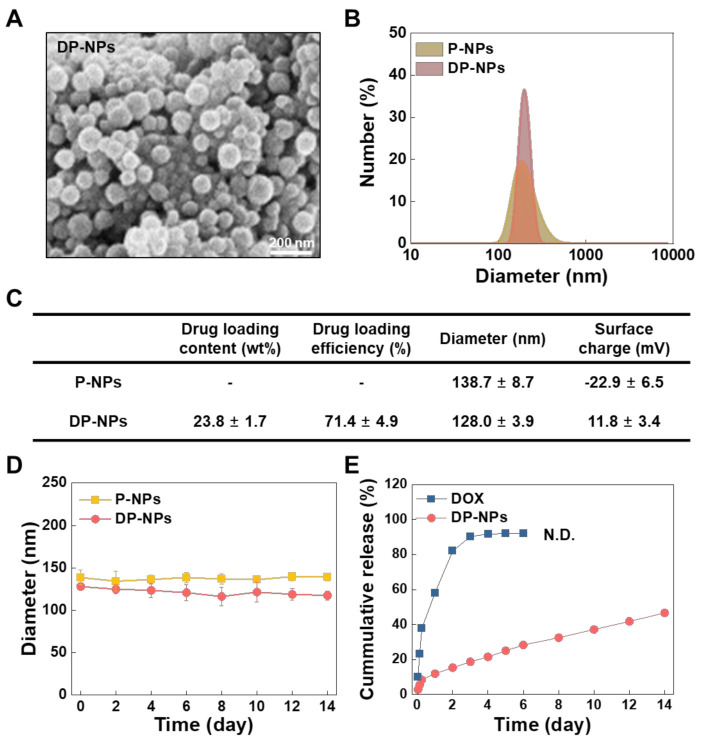
Physicochemical properties of the P-NPs and DP-NPs. (**A**) The morphology of the DP-NPs using FE-SEM. The scale bar indicates 200 nm. (**B**) The hydrodynamic size distribution of the P-NPs and DP-NPs using a zeta-sizer. (**C**) The DOX loading content and efficiency, particle size and surface charge of the P-NPs and DP-NPs using UV–vis spectroscopy and a zeta-sizer. (**D**) The size stability of the P-NPs and DP-NPs was monitored under 10% (v/v) FBS-containing PBS at 37 °C for 2 weeks using a zeta-sizer. (**E**) In vitro sustained release kinetics of DOX from the DP-NPs was monitored under 0.1% (v/v) tween 80-containing PBS at 37 °C for 2 weeks. The released amount of DOX was quantified using absorbance at 480 nm of UV–vis spectroscopy.

In this study, we prepared P-NPs by the o/w single-emulsion solvent evaporation method. DP-NPs were also prepared in the same way as P-NPs with the hydrophobically desalted DOX. Their formulation showed a loading efficiency of 23.8% in that the amount of encapsulated DOX in 1 mg of DP-NPs was measured 238 ± 17 μg by UV–vis spectroscopy. We also confirmed each unit of DP-NPs was spherical, and they had a smooth particle surface similar to P-NPs from SEM images (Figure 1A). Unimodal size distributions were observed in DP-NPs as well as P-NPs, wherein the average particle size of P-NPs and DP-NPs was 138.7 ± 8.7 and 128.0 ± 3.9 nm, respectively (Figure 1B). The surface charge of the P-NPs and DP-NPs was −22.9 ± 6.5 and 11.8 ± 3.4 mV, respectively, and this difference was mainly because of a surface charge change resulting from the encapsulation of the hydrophobically modified DOX into the P-NPs. Actually, the changes in surface charges were directly affected by the hydrophobicity/hydrophilicity of DOX. The characteristics of the P-NPs and DP-NPs were presented in Figure 1C. The particle size of the P-NPs and DP-NPs was stable in PBS with 10% FBS for 2 weeks (Figure 1D). The release behavior of DOX from DP-NPs was investigated for 2 weeks. DP-NPs dispersed in PBS (pH 7.4) with 0.1% (v/v) tween 80 were kept in a shaking incubator while maintaining a constant temperature (37 °C). Under these physiological conditions, a total of 46.6 ± 2.0% of DOX was released from the DP-NPs for 14 days (Figure 1E). This in vitro release study showed the encapsulated DOX had a short-term initial burst release within 6 h followed by a sustained release manner for almost 2 weeks. On the other hand, over nine-tenths of DOX was freed from DOX control, as opposed to one half of DOX was released from the DP-NPs. In summary, DP-NPs were successfully nanoformulated and spherical in shape with unimodal size distribution. They also showed the excellent serum stability for 2 weeks, with sustained drug release-rate kinetics.

### 3.2. In Vitro Cellular Uptake, Cytotoxicity, and ICD of DP-NPs

**Figure 2:**
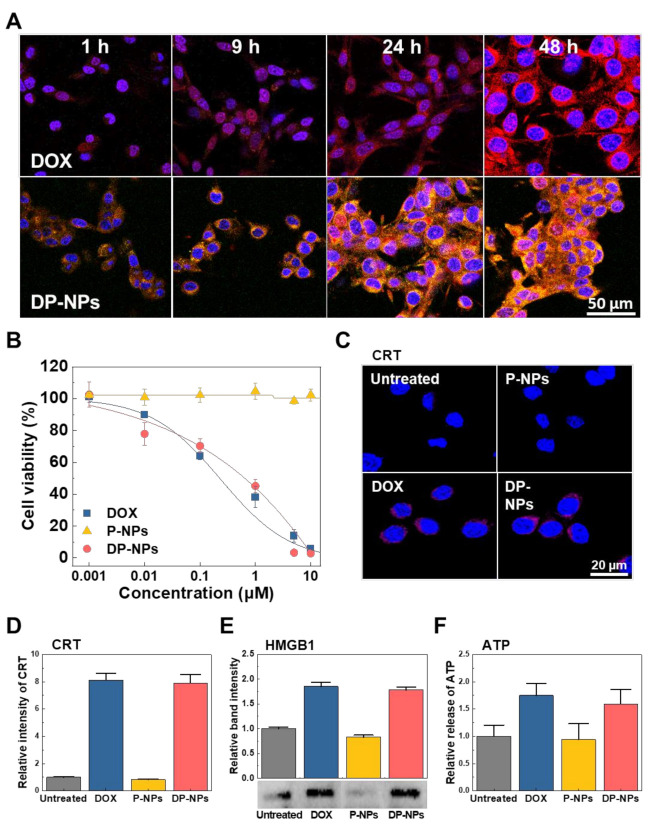
In vitro cellular uptake, cytotoxicity, and the subsequent ICD on the CT26 tumor cells. (**A**) Overlay confocal images of the time-dependent DOX-HCl and FITC-labeled-DP-NPs (2 μM of DOX) uptake. Blue: DAPI; Red: DOX; Green: PLGA. The scale bar indicates 50 μm. (**B**) Cell viability of DOX-HCl- and DP-NPs-treated CT26 tumor cells at increasing concentrations for 48 h using CCK-8. (**C**) Confocal images and (**D**) the quantified relative intensity of surface-exposed CRT in the DOX-HCl- and DP-NPs-treated (2 μM of DOX) CT26 tumor cells for 48 h. The scale bar indicates 20 µm. (**E**) Western blotting image and band intensity of released HMGB1 in the DOX-HCl- and DP-NPs-treated (2 μM of DOX) CT26 tumor cell culture medium for 48 h. (**F**) ATP level in the DOX-HCl- and DP-NPs-treated (2 μM of DOX) CT26 tumor cell culture medium for 48 h.

We used CT26 tumor cells to identify in vitro cellular uptake and localization of DOX, and to perform integrative analyses of the follow-on effects such as DOX-induced cytotoxicity and ICD. DOX-HCl and FITC-labeled DP-NPs (2 μM of DOX) were added to CT26 tumor cells and harvested from them right after the specific incubation time of 1 h, 9 h, 24 h, and 48 h, respectively, followed by detecting the signals of FITC and DOX fluorescence on time (Figure 2A). Specifically, green signals of FITC from the DP-NPs were increased over time in the cytoplasmic area of the CT26 tumor cells (Supplementary Materials Figure S1). In this sense, the cellular uptake of DP-NPs was shown to be successful by the incubation time. Meanwhile, there was a big difference in expression of red signals of DOX between DOX-HCl-treated and DP-NPs-treated CT26 tumor cells. From the red signal changes in the confocal images, fluorescence of DOX was largely detected in the nucleus at an incipient stage (at 1 h) in DOX-HCl-treated CT26 tumor cells, whereas it was gradually localized in the nucleus 24 h after the treatment of DP-NPs in CT26 tumor cells. Theretofore, DOX was mainly localized in the cytoplasmic area of CT26 tumor cells after the treatment of DP-NPs.

DOX-induced CT26 tumor cell cytotoxicity was determined by CCK-8 assay after the treatment of DOX-HCl and DP-NPs of which concentration ranged from 0.001 to 10 μM for 48 h (Figure 2B). The cell viability of both experimental groups was gradually decreased in proportion to the amount of the concentration of the treated DOX-HCl and DP-NPs; however, each group had somewhat different half maximal inhibitory concentration (IC_50_) values. In this study, the IC_50_ values obtained by CCK-8 assay against DOX-HCl- and DP-NPs-treated CT26 tumor cells were 0.227 μM and 0.641 μM, respectively. For reference, vehicle displayed no significant cytotoxicity, as if nothing was treated on the CT26 tumor cells. In comparison, the IC_50_ values of DP-NPs were almost threefold higher than that of DOX-HCl in CT26 tumor cells. The primary reason of this reduced cytotoxicity of DOX from DP-NPs was its sustained-release property.

Finally, from the CT26 tumor cells treated with DOX-HCl and DP-NPs, we assessed DOX-triggered DAMPs such as CRT, HMGB1, and ATP. CRT is protein that could translocate on the cell membrane of dying cells by the mechanism of ICD. To confirm the DOX-induced ICD, we observed the CRT translocation on the DOX-HCl- and DP-NPs (2 μM of DOX)-treated CT26 tumor cells for 48 h, using a confocal microscopy (Figure 2C). It was shown the fluorescence indicating surface-exposed CRT was significantly increased on the cell membrane when the CT26 tumor cells were treated with DOX-HCl and DP-NPs, compared to that of untreated and vehicle-treated CT26 tumor cells. Additionally, the relative fluorescence intensity of CRT from DOX-HCl- and DP-NPs-treated CT26 tumor cells was 8.12- and 7.91-fold higher than that of untreated CT26 tumor cells, respectively (Figure 2D). For reference, there were no significant differences in CRT levels between vehicle-treated and untreated CT26 tumor cells.

HMGB1 is a nuclear protein and also one of the ICD markers passively released from the dying cells. Similarly, to confirm the DOX-induced ICD, we observed the release of HMGB1 from the DOX-HCl- and DP-NPs (2 μM of DOX)-treated CT26 tumor cells for 48 h, and assessed by Western blotting. The band intensity indicating released HMGB1 was increased when the CT26 tumor cells were treated with DOX-HCl and DP-NPs, compared to that of untreated and vehicle-treated CT26 tumor cells (Figure 2E). Additionally, the relative band intensity of HMGB1 from DOX-HCl- and DP-NPs-treated CT26 tumor cells was 1.85- and 1.78-fold higher than that of untreated CT26 tumor cells, respectively. For reference, there were no significant differences in HMGB1 release between vehicle-treated and untreated CT26 tumor cells.

ATP is one of the ICD markers and a molecule emitted actively from cytoplasm and cytoplasmic organelles in dying cells. The secretion of ATP from the DOX-HCl- and DP-NPs (2 μM of DOX)-treated CT26 tumor cells for 48 h, was determined by the ATP assay to confirm the DOX-induced ICD. The measured levels indicating secreted ATP was increased when the CT26 tumor cells were treated with DOX-HCl and DP-NPs, compared to that of untreated and vehicle-treated CT26 tumor cells (Figure 2F). Additionally, the relative level of ATP from DOX-HCl- and DP-NPs-treated CT26 tumor cells was 1.75- and 1.59-fold higher than that of untreated CT26 tumor cells, respectively. For reference, there were no significant differences in ATP secretion between vehicle-treated and untreated CT26 tumor cells.

We were able to confirm that the diverse aspects of DAMPs, including CRT translocation and release of HMGB1 and ATP, were very closely correlated with the cell viability after the treatment of DOX. Based on these results, it was expected that DP-NPs could sufficiently induce DAMPs-mediating ICD to CT26 tumor cells via time-releasing DOX.

### 3.3. In Vivo Tumor Growth Inhibition by DP-NPs-Driven CIT

**Figure 3:**
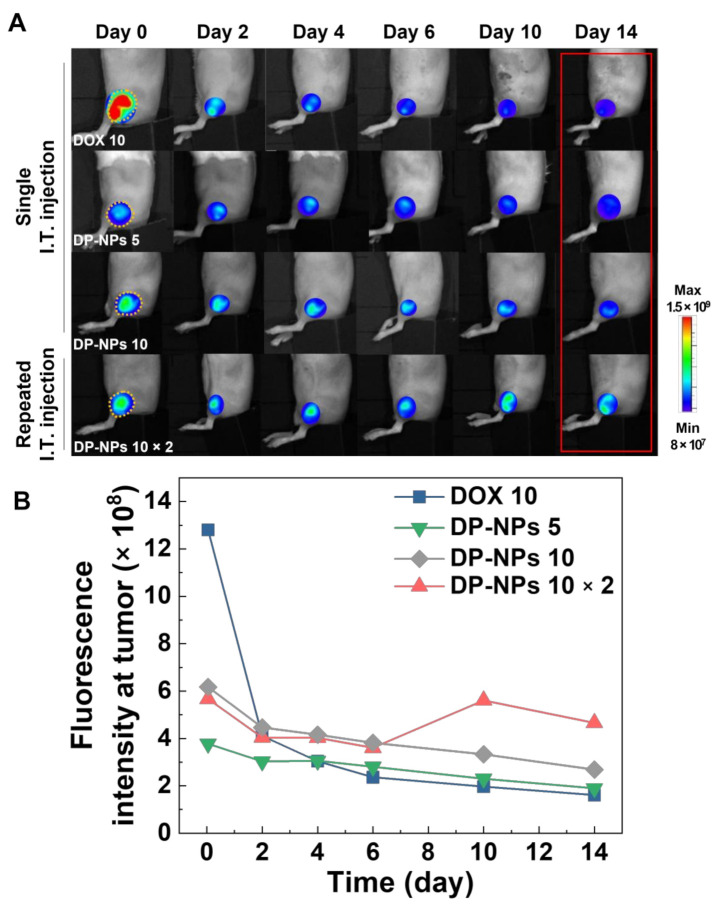
In vivo drug release characteristics of DP-NPs after their intratumoral injection. (**A**) Time-dependent fluorescence images of the CT26 tumor-bearing mouse intratumorally injected with DOX-HCl (10 mg/kg) and DP-NPs (5, 10, 10 × 2 mg/kg) for 2 weeks. (**B**) Quantified fluorescence intensity of DOX at the tumor tissues standing on the basis of the images from (**A**).

To observe in vivo release kinetics of DOX from DP-NPs, we obtained time-dependent fluorescence images by non-invasive optical imaging system, visualizing the accumulated DOX in targeted tumor tissues. In order to find the optimal drug concentration, fulfilling therapeutic window of DOX and also maximizing therapeutic efficacies, we set up several experiments with groups of various concentration of DP-NPs. In this regard, we tracked the fluorescence signals of DOX released from the DP-NPs at the targeted tumor tissues for 14 days, after the intratumoral injection of DP-NPs (5, 10, 10 × 2 mg/kg) (Figure 3A). DOX-HCl (10 mg/kg) was used as a control to compare the other groups of DP-NPs. DOX fluorescence in the DOX-HCl-treated CT26 tumor-bearing mice was extremely decreased to the level in 10 mg/kg of DP-NPs-treated group in just 2 days, and since then, it has continued to decrease. On the other hand, DP-NPs-treated groups showed mild decrease in proportion to their initial DOX concentration without a wide variation of fluorescence levels. Notably, the DOX fluorescence at the tumor site of DP-NPs 10 × 2-treated group showed gradually decreased for 6 days but recovered by second intratumoral injection, resulting in maintaining the DOX fluorescence range for 14 days. In short, there were different patterns of the change in relative DOX fluorescence intensity at the tumor that were drastically decreased, continuously decreased, or maintained a certain range of fluorescence after the initial ascending (Figure 3B). From these findings, we confirmed PK profile of DOX could be favorably controlled by sustained-released DOX from the directly injected DP-NPs. DP-NPs were confined to the tumor tissues within a given period, and DOX was successfully supplied locally, especially in the group of repeated treatment of DP-NPs. This result sufficiently gave us a sound basis for the continuous ICD and the subsequent antitumor immune responses.

**Figure 4:**
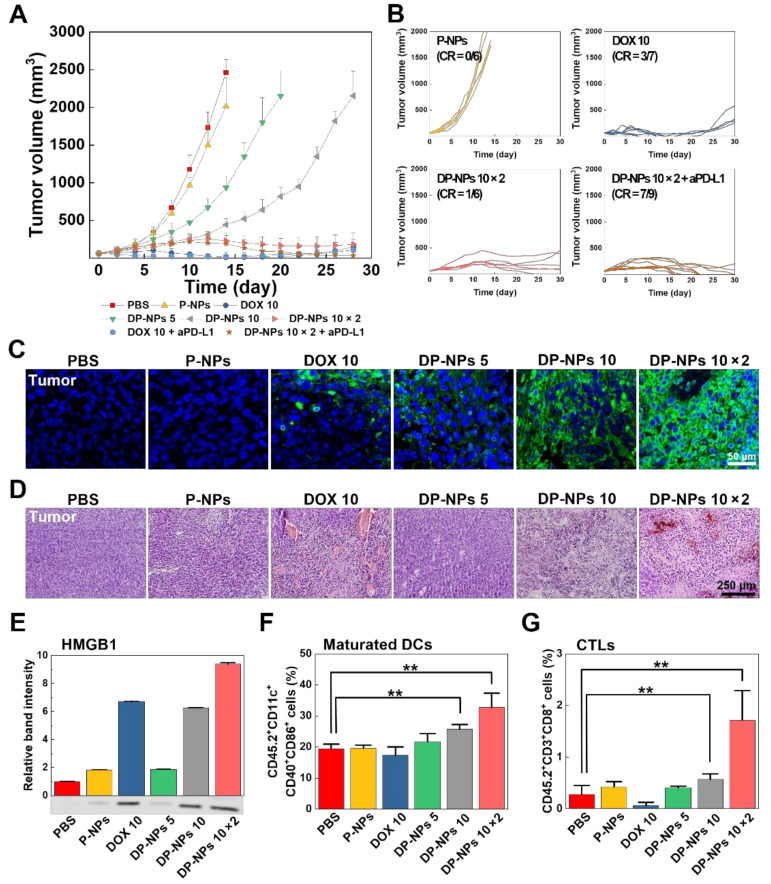
In vivo tumor growth inhibition and the subsequent antitumor immune responses. (**A**) Tumor growth of the CT26 tumor-bearing mice after the first intratumoral injection of PBS, P-NPs, DOX-HCl (10 mg/kg), DP-NPs (5, 10, 10 × 2 mg/kg), aPD-L1 Ab (10 × 5 mg/kg)-combined DOX-HCl (10 mg/kg), and DP-NPs (10 × 2 mg/kg) for 4 weeks. (**B**) Tumor growth for individual mice and CR rate in P-NPs-, DOX-HCl-, DP-NPs-, and aPD-L1 Ab-combined DP-NPs-treated groups of (**A**). Representative images of (**C**) TUNEL- and (**D**) H&E-stained tumor tissues at 14 days after the first treatment of PBS, P-NPs, DOX-HCl (10 mg/kg), and DP-NPs (5, 10, 10 × 2 mg/kg). TUNEL-positive cells are visible as green. The scale bars indicate 50 and 250 µm, respectively. (**E**) Western blotting image and band intensity of in vivo HMGB1 at 14 days after the first treatment of PBS, P-NPs, DOX-HCl (10 mg/kg), DP-NPs (5, 10, 10 × 2 mg/kg). Immune cell population of (**F**) maturated DCs in TDLNs and (**G**) CTLs in tumor tissues at 14 days after the first treatment of PBS, P-NPs, DOX-HCl (10 mg/kg), and DP-NPs (5, 10, 10 × 2 mg/kg), analyzed using a flow cytometer. Double asterisks (**) indicate a difference at the *p* < 0.01 significance level.

We confirmed the effects of DOX-based CIT and its induced TIME changes in the CT26 tumor-bearing mice model. Tumor growth of the CT26 tumor-bearing mice treated with DOX-HCl (10 mg/kg), DP-NPs (5, 10, 10 × 2 mg/kg of DOX) was measured for 4 weeks after their first intratumoral injections (Figure 4A). Injection dose of each group was the same as in the previous in vivo imaging experiment, and as described earlier, 10 × 2 mg/kg of DP-NPs was injected in twice with an interval of 10 days. For reference, PBS and P-NPs were used as controls, and we limited the maximum allowable size of subcutaneous CT26 tumors (2000 mm^3^ volume in mice) in these control experimental groups. DOX-HCl (10 mg/kg; n = 7) strongly inhibited tumor growth up to 4 weeks, and the average tumor size of DOX-HCl-treated mice was 150 mm^3^ on the 28th day. Meanwhile, DP-NPs (10 × 2 mg/kg; n = 6) exhibited effective tumor suppression as compared with PBS (n = 5) and P-NPs (n = 6), and also apparently it was better than DP-NPs of different doses (5, 10 mg/kg; n = 6, 5, respectively). The average tumor size of DP-NPs (10 × 2 mg/kg)-treated mice was 184 mm^3^ on the same day above, and the size of all the others were over 2000 mm^3^.

In addition to these results, we also used an additional aPD-L1 Ab for synergistic combination cancer CIT to elicit the better antitumor therapeutic effects. aPD-L1 Ab is one of the widely used ICBs in cancer immunotherapy. Engagement of PD-L1 blockade by PD-L1 expressed on tumor cells leaded to the enhancement of tumor-specific T cell-mediated adaptive immunity. Based on the ICB effect and enhancing adaptive immunity, furthermore, recent studies showed that the tumor progression was significantly inhibited by combination with PD-L1 blockade therapy, indicating the therapeutic potential for cancer CIT. Based on these hard facts with the evidence of our previous findings, PD-L1 blockade combined with DOX-HCl and DP-NPs treatment were carried out to evaluate therapeutic outcomes, respectively. Tumor growth of the CT26 tumor-bearing mice of these two groups were monitored for 4 weeks. 10 mg/kg of aPD-L1 Ab was administered to mice of all combination groups five times every 3 days via the intraperitoneal route (Supplementary Materials Figure S2). According to the previously stated results, tumor gradually regrew in DOX-HCl (10 mg/kg)-treated group, whereas the tumor in DP-NPs (10 × 2 mg/kg)-treated group was well-circumscribed with an average volume under 200 mm^3^, approximately 2 weeks after their first treatments. In these combination study, tumor also gradually regrew in DOX-HCl (10 mg/kg) plus aPD-L1 Ab (10 × 5 mg/kg)-treated group 18 days after the first DOX-HCl injection, similarly in DOX-HCl-treated group. Interestingly, the extremely inhibited tumor growth was observed in DP-NPs (10 × 2 mg/kg) plus aPD-L1 Ab (10 × 5 mg/kg) group. The average tumor volume in this group gradually decreased and did not exceed 50 mm^3^, 24 days after the first DP-NPs injection.

Next, we analyzed the complete response (CR) rate of each treatment based on the tumor size on the 28th day (Figure 4B and Supplementary Materials Figure S3). Generally, the term CR applies in case that all existed tumor is no longer observed after the treatment. This CR rate of DOX-HCl- and DP-NPs (10 × 2 mg/kg)-treated groups was 42.9% and 16.7%, respectively. It was shown the other DP-NPs (5, 10 mg/kg)-treated groups just partially responded to the treatment, and there were no tumor-suppressed mice in controls such as PBS- or P-NPs-treated groups. However, PD-L1 blockade-combined DP-NPs (10 × 2 mg/kg) exhibited highly increased rate of CR, compared to DOX-HCl (10 mg/kg) with PD-L1 blockade. The specific CR rate of these groups was 77.8% and 40.0%, respectively. It was shown therapeutic efficacies in ICB combination tended to correlate with reduction in tumor growth, especially in PD-L1 blockade-combined DP-NPs-treated group.

We could obtain factual basis of tumor inhibition by DOX from TUNEL assay. DOX-HCl and DP-NPs had apoptotic effects on the tumor tissues in each group, leading to the loss of tumor cell viability. The number of TUNEL-positive tumor cells were highly distributed in these groups, and it was increased as the concentration of DOX was increased (Figure 4C). The reason of significantly increased levels of TUNEL-positive cells in DP-NPs-treated tumor tissues, especially in 10 × 2 mg/kg of DP-NPs-treated tissues, was mainly considered as sustained drug release properties of DP-NPs. We also observed tissue morphologies and its structural abnormalities by histology from the H&E-stained tumor tissues harvested on the 14th day after the first intratumoral treatment in each group (Figure 4D). It was identified CT26 tumor tissues from PBS- or P-NPs-treated mice had few or no necrotic areas, and they displayed less or no toxicity. On the contrary, there was a large necrotic area over the tumor tissues from DOX-HCl-treated mice, indicating high toxicity was caused by DOX. There were also analogical results along the above, tumor tissues from DP-NPs-treated mice showed several similar histotoxicities, however different by their dose. It was shown higher amounts of localized DP-NPs could release a lot more DOX, actually leading to certain levels of toxicity and histological changes in TME.

Subsequently, we confirmed the immunological evidences in TME correlated with the previous results. As previously stated, ICD is generally known as an initial key process in antitumor immunity, it directly becomes a link between subsequent innate immunity and adaptive immunity. In this regard, to measure the HMGB1 levels from chemotherapeutics-treated CT26 tumor cells in vivo, we observed the release of HMGB1 in each group treated with DOX-HCl and DP-NPs with different dose levels, and assessed by Western blotting on their 14th day of our treatment. As expected from the previous in vitro results, these in vivo tests also confirmed the measurements of elevated levels of the band intensity indicating released HMGB1 in DOX-HCl- and DP-NPs-treated groups. Specifically, the relative band intensity of HMGB1 from DOX-HCl (10 mg/kg)- and DP-NPs (10, 10 × 2 mg/kg)-treated groups was 6.71-, 6.27-, and 9.39-fold higher than that of PBS-treated group, respectively (Figure 4E). For reference, there were no significant differences in HMGB1 level between vehicle-treated group and PBS-treated group. Especially, as shown above, exceedingly strong ICD was induced in the tumor tissues and TME of the DP-NPs (10 × 2 mg/kg)-treated group, in contrast with the results of controls and even DP-NPs (5 mg/kg)-treated group. This 5 mg/kg of DP-NPs was not seemed to be enough to induce and maintain the ICD continuously in vivo.

DOX-triggered ICD induce ICD-associated DAMPs in TME, thereby activating innate immunity represented by DC maturation, leading to efficient priming of CTLs, eventually resulting in effective antitumor immunity with newly changed TIME. To verify the immunological basis for this, DCs and CTLs isolated from TDLNs and tumor tissues in the CT26 tumor-bearing mice in each experimental group were examined by flow cytometry. These analyses were performed on the 14th day after the first intratumoral injection of PBS, P-NPs, DOX-HCl (10 mg/kg), and DP-NPs (5, 10, 10 × 2 mg/kg). According to our analytical results, populations of maturated DCs were high in DP-NPs-treated groups, and this population value was gradually increased as a dose of treated DP-NPs (Figure 4F). The value was the highest in 10 × 2 mg/kg of DP-NPs-treated group, wherein strongly induced ICD continuously enhanced maturation of DCs in TDLNs. On the other hand, DP-NPs-treated groups showed extremely higher CTL populations than DOX-HCl-treated group (Figure 4G). This result was rooted in whether CTLs were directly exposed to DOX or not in that CTLs were reckoned to be vulnerable to DOX-induced cytotoxicity.

Summarizing the results so far, DP-NPs successfully suppressed tumors in vivo, as shown in the results of effectively inhibited growth of CT26 tumors. It seemed we could sufficiently control the ICD being induced and maintained continuously in vivo by over a certain amount of DOX. Meanwhile, a high initial concentration of DOX in the DOX-HCl-treated group could induce severe toxic effects on major organs as well as immune cells including maturated DCs and CTLs. Furthermore, it was also identified that strongly induced ICD was not connected to adaptive immunity. Nonetheless, these results were significant in the sense that CD8-positive CTLs primed to the TIME favorably acted against the tumor due to the sustained-released DOX from the DP-NPs, even in high doses of DP-NPs (10 × 2 mg/kg) with reduced toxicity and much stronger ICD, leading to effective therapeutic results. Additionally, it was considered largely populated CTLs in TIME favorably killed the tumor cells better in the combination therapy group with enhanced antitumor immunity, attributable to the help of ICB.

**Figure 5:**
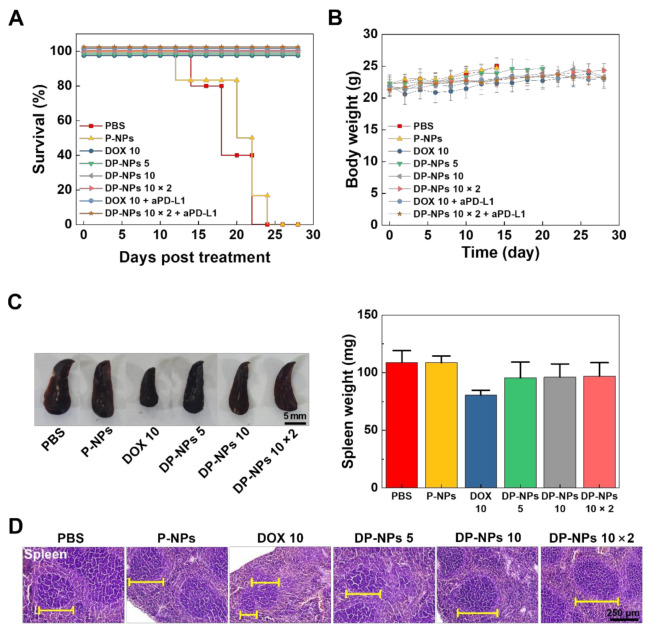
In vivo DOX-induced toxicity in the BALB/c mouse model. (**A**) Survival rate and (**B**) body weight change of the CT26 tumor-bearing mice after the first intratumoral injection of PBS, P-NPs, DOX-HCl (10 mg/kg), DP-NPs (5, 10, 10 × 2 mg/kg), aPD-L1 Ab (10 × 5 mg/kg)-combined DOX-HCl (10 mg/kg), and DP-NPs (10 × 2 mg/kg) for 4 weeks. (**C**) Representative photos and the weight of spleens from the CT26 tumor-bearing mice treated with PBS, P-NPs, DOX-HCl (10 mg/kg), DP-NPs (5, 10, 10 × 2 mg/kg) at 14 days after the first treatments. The scale bar indicates 5 mm. (**D**) Representative images of H&E-stained spleen tissues at 14 days after the first treatment of PBS, P-NPs, DOX-HCl (10 mg/kg), DP-NPs (5, 10, 10 × 2 mg/kg). Yellow line indicates the spleen white pulp (dark blue) in the H&E-stained histological images. The scale bar indicates 250 µm.

For survival, mice with a tumor size 3000 mm^3^ were counted as statistically dead. In this regard, PBS- or P-NPs-treated groups showed big changes in animal survival for 4 weeks (Figure 5A). Body weights of mice in each group were monitored with tumor volumes every 2 days. It was considered there were no severe systemic toxicities during the treatment in that there were no big differences in body weight change (Figure 5B). However, spleen weight of the DOX-HCl-treated mice was decreased by more than 20%, compared to that of PBS- or P-NPs-treated mice (Figure 5C). Furthermore, the tissue morphology of shrunken spleen from the DOX-HCl-treated mice showed a decreased size of white pulp and larger red pulp, compared to those of PBS-, P-NPs-, and DP-NPs (5, 10, 10 × 2 mg/kg)-treated mice. We expected that the shrinking and morphological changes of the spleen occurred because of the undesirable toxicity of DOX-HCl, resulting in underperforming normal function (Figure 5D) [44]. Additionally, intratumorally injected DP-NPs (5, 10, 10 × 2 mg/kg) also exhibited relatively less systemic toxicities in the spleen and other organs (liver, lung, kidney, and heart) rather than DOX-HCl, based on the histopathology data (Supplementary Materials Figure S4). From these results, exposure to therapeutic concentration levels of DOX (even in case of 10 × 2 mg/kg of DOX) was acceptable in some cumulative toxicity burdens by using our nanoparticulate formulation of DP-NPs.

## 4. Conclusions

Chemotherapeutics are widely used and in cancer immunotherapy, and as is well known, they initiate immunogenic antitumor reactions and contribute to promoting the antitumor immunity by facilitating the infiltration of immune cells into the tumor [45]. However, uncontrolled high-dose chemotherapy can immoderately generate cytotoxic reaction and immunosuppression in TME, inevitably resulting in the negative effects on the functions of immune effectors [46]. In this sense, the sustained-release nanomedicine is necessarily required in cancer immunotherapy, especially to use the chemotherapeutics bio- and immuno-compatibly. In this study, we developed DP-NPs that could sustain the release of DOX for controlling ICD with toxicity in the TIME of the tumor tissues. Nano-sized DP-NPs released DOX for 14 days under physiological conditions in vitro. Furthermore, released DOX successfully induced ICD on the CT-26 tumor cells, resulting in significantly decreasing tumor cell viability. Intratumorally injected DP-NPs enabled prolonged retention of DOX for 14 days, compared to that of DOX-HCl. Concurrently, we confirmed the maximized therapeutic outcomes for 4 weeks created by the time-releasing DP-NPs and ICB combination effect. The continuous ICD in the tumor by DP-NPs 6.27 to 9.39-fold higher HMGB1 release than that of PBS-treated tumor tissues led to higher DC maturation and elicited a higher proportion of CTLs into tumor tissues. Furthermore, the DP-NPs-treated mice showed less toxicities in immune cells and spleen than those of DOX-HCl treated mice during CIT. Finally, we expect that combination CIT using DP-NPs and ICB will reconstitute TIME for the augmented antitumor immunity and control toxicity at the same time, can provide therapeutic strategies for the best therapeutic results.

## Data Availability

The data presented in this study are available upon request from the corresponding author.

## Supplementary Materials

The following are available online at https://www.mdpi.com/article/10.3390/pharmaceutics14030474/s1. Figure S1: Individual images of Figure 2A. Blue: DAPI; Red: DOX; Green: PLGA. The scale bar indicates 50 μm, Figure S2: Time schedule of the whole treatment processes for CT26 tumor in BALB/c mice, Figure S3: Tumor growth for individual mice and CR rate in PBS-, DP-NPs (5, 10 mg/kg)-, and aPD-L1 Ab (10 × 5 mg/kg)-combined DOX-HCl (10 mg/kg)-treated groups of Figure 4A, Figure S4: Representative images of H&E-stained tissues (liver, lung, kidney, and heart) at 14 days after the first treatment of PBS, P-NPs, DOX-HCl (10 mg/kg), DP-NPs (5, 10, 10 × 2 mg/kg). The scale bar indicates 250 µm.
