# Supplementary material for: Sustained and Long-Term Release of Doxorubicin from PLGA Nanoparticles for Eliciting Anti-Tumor Immune Responses
Source: Pharmaceutics. 2022 Feb 22;14(3):474. doi: 10.3390/pharmaceutics14030474 (PMC8954063; doi:10.3390/pharmaceutics14030474)
Supplement: Supplementary file 1 [file pharmaceutics-14-00474-s001.zip › pharmaceutics-1595469-supplementary.pdf]

Supplementary materials

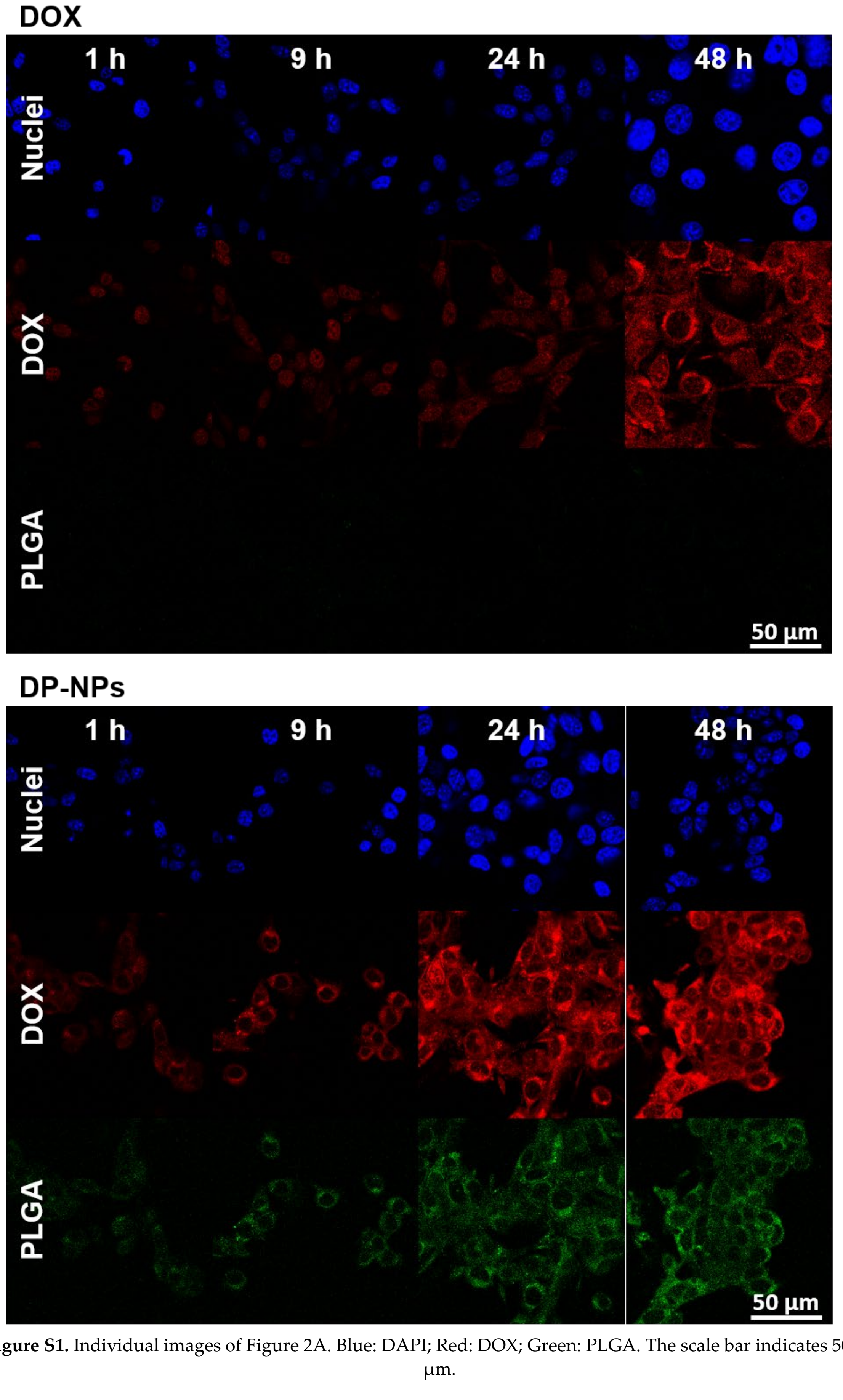

Figure S1. Individual images of Figure 2A. Blue: DAPI; Red: DOX; Green: PLGA. The scale bar indicates 50 µm.

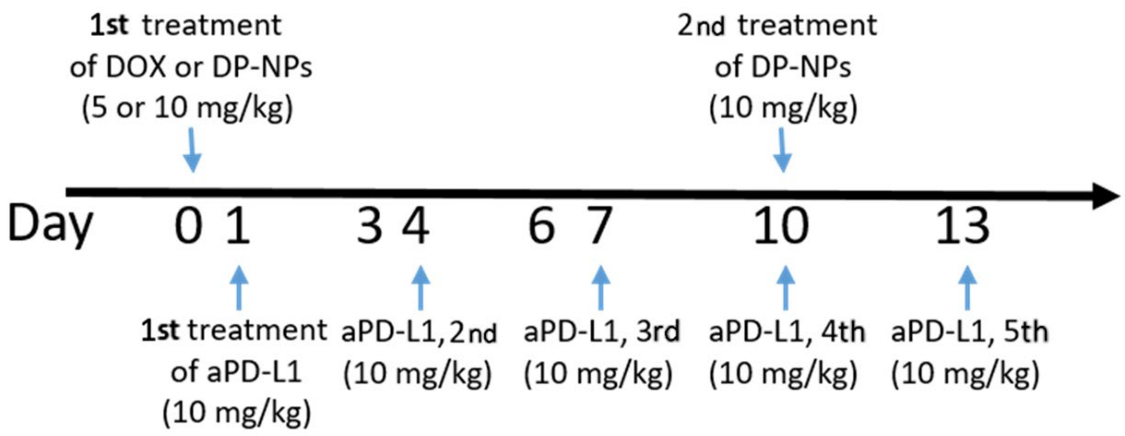

Figure S2. Time schedule of the whole treatment processes for CT26 tumor in BALB/c mice.

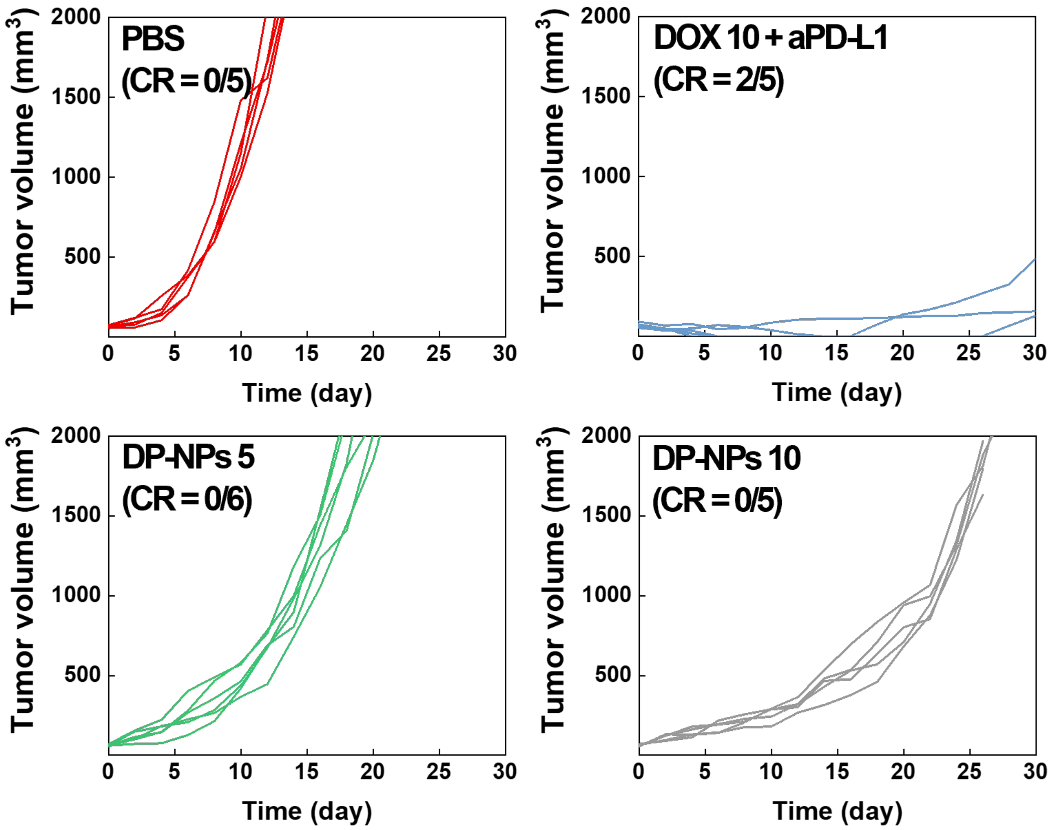

Figure S3. Tumor growth for individual mice and CR rate in PBS-, DP-NPs (5, 10 mg/kg)-, and anti-PD-L1 Ab (10 × 5 mg/kg)-combined DOX-HCl (10 mg/kg)-treated groups of Figure 4A.

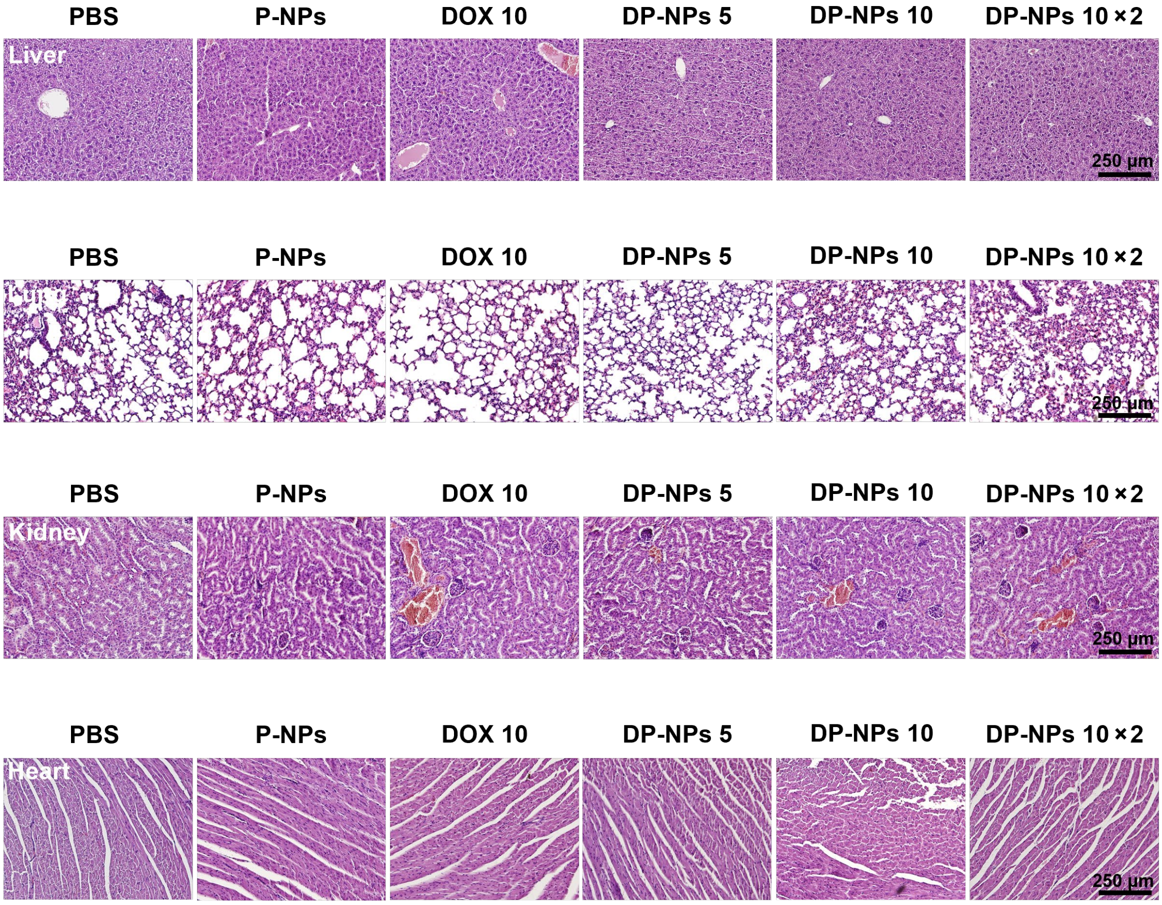

Figure S4. Representative images of H&E-stained tissues (liver, lung, kidney, and heart) at 14 days after the first treatment of PBS, P-NPs, DOX-HCl (10 mg/kg), DP-NPs (5, 10, 10*2 mg/kg). The scale bar indicates 250 μm.
